# Supplementary material for: I-Cubid: a nonlinear cubic graph-based approach to visualize and in-depth browse Flickr image results
Source: PeerJ Comput Sci. 2023 Aug 10;9:e1476. doi: 10.7717/peerj-cs.1476 (PMC10496001; doi:10.7717/peerj-cs.1476)
Supplement: Supplemental Information 1 [file peerj-cs-09-1476-s001.pdf]

Table 1: User evaluation consent form.

☐ I understand that this evaluation is used for purely research purposes.
☐ I understand that my participation is voluntarily and I Can withdraw if unwilling.
☐ I understand that my personal information such as name will be kept private.
☐ I understand that by writing my name in evaluation demographic form I give my consent to carry out I-Cuboid research evaluation process.

Appendix A. User Consent, Demography, Evaluation Tasks, Raw SUS and QUIS Scores

Table 2: Required users' demographic details.

#	Age (Years)	Gender (M/F)	Occupation	Image Search Activity Time per Week	Image Search Experience (Years)	Preferred Image Search Tool	Topic
1	27	F	PhD Student + Teacher	Less than 1H	15	Google, Facebook	3
2	26	F	MPhil Student	More than 9H	10	Google, Instagram, Facebook	2
3	25	F	MPhil Student	More than 9H	15	Google, Bing, PhotoBucket	2
4	25	F	Student + Housewife	Less than 1H	8	Google	4
5	45	M	Govt. Servant	Less than 1H	10	Google	2
6	43	F	Housewife	Less than 1H	Nil	Google	3
7	23	M	Under-Grad Student	Less than 1H	4	Google	4
8	21	F	Under-Grad Student	1-3H	5	Google, Instagram	1
9	22	F	Under-Grad Student	More than 9H	6	Google	1
10	23	M	Under-Grad Student	Less than 1H	7	Google	2
11	32	M	IT Support	Less than 1H	5	Flicker	3
12	27	M	App Developer	1-3H	10	Google, Bing	4
13	32	F	Home Tutor	1-3H	15	Google	1
14	28	M	Researcher	1-3H	5	Google	4
15	30	M	Software Developer	None	Nil	Personal Photo Folder	3
16	26	M	Software Engineer	1-3H	4	Google	2

Table 3: Evaluation topics and tasks detail.

Type	Topic	Goldfish in Aquarium	Bullet Trains in Japan	Fighter Aircraft in Pakistan	Indian Tiger
Guide Task	Step	Grid-based Presentation		Cube-based Presentation	
	1	Input query {topic}		Input query {topic}	
	2	Select an image in seventh row and third column on the grid.		Select second plane from the cube.	
	3	Select image in third row and fourth column of second plane.		Select image in third row and fourth column of second plane.	
	4	View details of the selected image.		View details of the selected image.	
	5	Download the selected image on your computer.		Download the selected image on your computer.	
	6	View the neighbors (left, right, top, and bottom) of the selected image.		View the neighbors (left, right, top, bottom, next, previous) of the selected image.	
	7	On the second page, select image in thirteenth row and sixth column.		Select any image from the neighbor images.	
	8	View details of the selected image.		View details of the selected image.	
	9	Download the selected image on your computer.		Download the selected image on your computer.	
Unguided Task	1	Search {topic}		Search {topic}	
	2	Identify a pair of entities related to {topic} view its details, and download it on your computer from the first page.		Identify pair of entities related to {topic} by exploring all the planes of the cube, view the details of the identified image, and download it on your image.	
	3	Explore the neighbor images of the selected image.		Explore the neighbor images of the selected image.	
	4	Select the next page by clicking the next button.		Select the next cube by clicking the next button.	
	5	Identify an image containing a pair entity related to {topic} with white background, view its details, and download it on your computer from the second page.		Identify an image containing a pair entity related to {topic} with white background, view the details of the identified image, and download it on your computer.	
	6	Explore the neighbor images of the selected image.		Explore the neighbor images of the selected image.	

Table 4: Grid presentation system usability results.

User/Q.No	Q1	Q2	Q3	Q4	Q5	Q6	Q7	Q8	Q9	Q10	Sum	Score Value
1	4	2	4	2	4	2	4	2	4	3	29	72.5
2	5	2		3	5	3	5	1	3	1	28	70
3	1	2	3	2	3	3	1	1	4	3	21	52.5
4	3	3	3	2	3	2	2	2	4	2	24	60
5	3	2	4	1	2	2	4	2	5	1	30	75
6	4	1	4	3	5	1	5	1	5	4	33	82.5
7	5	1	5	1	5	1	5	1	5	1	40	100
8	5	1	5	1	4	1	5	1	4	1	38	95
9	4	1	5	3	4	2	5	1	5	2	34	85
10	4	2	5	1	4	3	4	2	5	1	33	82.5
11	4	2	1	2	2	2	1	2	4	2	22	55
12	5	4	5	5	4	3	3	1	5	1	28	70
13	4	1	5	2	5	2	5	1	4	1	36	90
14	4	1	5	1	5	1	5	1	5	1	39	97.5
15	4	3	4	1	4	1	3	2	4	2	30	75
16	5	1	5	1	5	1	5	1	5	1	40	100
17	5	1	5	1	5	1	5	1	5	2	39	97.5
18	4	1	5	1	5	2	5	1	5	1	38	95
Average Score												80.8

Table 5: Cube presentation system usability scale results.

User/Q.No	Q1	Q2	Q3	Q4	Q5	Q6	Q7	Q8	Q9	Q10	Sum	Score Value
1	4	2	4	2	4	2	4	2	4	3	29	72.5
2	4	3	5	4	4	3	4	2	3	3	25	62.5
3	5	1	1	5	5	1	5	2	5	3	29	72.5
4	3	2	4	2	4	2	4	2	4	2	29	72.5
5	5	1	5	1	5	1	5	1	5	1	40	100
6	2	4	2	1	2	4	3	2	4	4	18	45
7	5	1	5	1	5	1	5	1	5	1	40	100
8	5	1	5	1	5	1	4	1	4	1	38	95
9	4	5	3	4	3	4	5	2	5	2	23	57.5
10	5		5	2	4	3	4	3	5	2	33	82.5
11	5	1	5	3	5	1	4	1	5	2	36	90
12	5	4	4	4	5	3	3	3	5	2	26	65
13	4	3	4	3	2	2	4	4	3	4	21	52.5
14	3	2	3	1	4	1	5	2	4	2	31	77.5
15	4	2	4	2	3	2	3	2	4	2	28	70
16	5	1	5	1	5	1	5	1	5	1	40	100
17	5	1	5	2	5	1	5	1	5	1	39	97.5
18	4	1	5	1	5	2	5	1	5	1	38	95
Average Score												78.2

Table 6: Grid presentation questionnaire for user interfaces satisfaction results.

**User/Type**	**Overall**	**Screen**	**Sys. Info. & Ter.**	**Learning**	**Sys. Cap.**	**Average**	**Score Value**
1	8	8	8	7.7	8	7.9	88.1
2	8.4	8.3	7	8.2	6	7.6	84.2
3	5.5	3.8	4.8	4	4.4	4.5	50.1
4	6.7	6.3	6.7	6	4.2	6.0	66.3
5	6.8	5.5	6.2	7.7	7.8	6.8	75.3
6	9	8.8	9	9	9	9.0	99.6
7	9	9	9	9	9	9	100
8	8.3	9	7.3	8.8	9	8.5	94.4
9	8.7	8	5.7	8	8	7.7	85.2
10	8.3	8.3	7.8	8.7	8.8	8.4	93.2
11	1.2	2.8	2.3	3.3	1	2.1	23.7
12	7.7	6.3	8	8.5	6	7.3	81
13	8.7	8	8	8.5	8.3	8.3	92
14	6.8	9	9	9	9	8.6	95
15	7	7	7.5	7.3	7.4	7.3	80.56
16	9	9	8	8	8	8.4	93.3
17	8.8	8.5	8.8	8.8	8.8	8.8	97.2
18	8.5	8.8	7.75	9	8.2	8.5	93.9
Average	7.6	7.5	7.3	7.8	7.3	7.5	83.0
Score Value	84.1	83	80.8	86.1	80.8	83	83.0

Table 7: Cube presentation questionnaire for user interface satisfaction results.

**User/Q.No**	**Overall**	**Screen**	**Sys. Info. & Ter.**	**Sys. Info. & Ter.**	**Sys. Cap.**	**Average**	**Score Value**
1	6.8	7.3	7.0	7.5	8.0	7.3	81.3
2	7.4	8.0	6.3	6.8	5.4	6.8	75.3
3	5.5	7.3	8.3	8.0	7.6	7.3	81.6
4	7.2	7.5	7.6	6.8	6.0	7.0	77.8
5	8.5	8.5	8.2	9.0	9.0	8.6	96.0
6	1.3	2.3	2.3	2.0	5.8	2.7	30.4
7	9.0	9.0	9.0	9.0	9.0	9.0	100.0
8	8.7	9.0	7.6	9.0	9.0	8.7	96.1
9	6.0	7.7	8.5	4.0	2.0	5.6	62.6
10	8.3	8.3	8.2	8.5	8.4	8.3	92.6
11	9.0	9.0	9.0	9.0	9.0	9.0	100.0
12	7.3	7.3	7.7	8.0	6.8	7.4	82.2
13	6.7	5.0	6.0	5.2	7.0	6.0	66.3
14	7.0	6.7	9.0	8.8	9.0	8.1	89.8
15	7.7	7.3	7.3	6.7	7.2	7.2	80.2
16	9.0	9.0	8.0	8.0	8.0	8.4	93.3
17	8.8	9.0	8.8	8.7	8.8	8.8	98.0
18	8.5	8.8	8.0	9.0	8.2	8.5	94.3
Average	7.4	7.6	7.6	7.4	7.5	c 7.5	c 83.2
Score Value	81.9	84.3	84.6	82.6	82.8	83.2	c 83.2
