# Supplementary material for: I-Cubid: a nonlinear cubic graph-based approach to visualize and in-depth browse Flickr image results
Source: PeerJ Comput Sci. 2023 Aug 10;9:e1476. doi: 10.7717/peerj-cs.1476 (PMC10496001; doi:10.7717/peerj-cs.1476)
Supplement: Supplemental Information 2 — The raw SUS and QUIS usability scores for the Cube and Grid interfaces. This also includes the detailed demonstration view, search tasks and queries used for experimental purposes. [file peerj-cs-09-1476-s002.zip › I-Cuboid Evaluation Material/I-Cuboid Evaluation Material/Questionaire/Demographic Details and Consent.docx]

**Demographic Details and Consent**

**Name:** _____________________________________________________________________________

**Age (Years):** ________________________________________________________________________

**Gender:**

- Male
- Female

**Occupation:** ________________________________________________________________________

**Image Search Activity Time in Week**

- Less than 1 Hour
- 1-3 Hours
- 4-6 Hours
- 7 – 9 Hours
- More than 9 Hours

**Image Search Experience (Years):** ____________________________________________________

**Tool Used to Search Images**

- Google
- Bing
- Flicker
- Yahoo
- Instagram

Any other: ___________________________________________________________________________

**Choose Topic:**

- Gold Fish in Aquarium
- Bullet Trains in Japan
- Fighter Aircraft in Pakistan
- Indian Tiger

I understand that this evaluation is used for purely research purposes.

I understand that my participation is voluntarily, and I can withdraw If unwilling.

I understand that my personal information such as name, will be kept private.

I understand that by writing my name in evaluation demographic form, I give my consent to carry out I-Cubid research evaluation process.
