# Supplementary material for: I-Cubid: a nonlinear cubic graph-based approach to visualize and in-depth browse Flickr image results
Source: PeerJ Comput Sci. 2023 Aug 10;9:e1476. doi: 10.7717/peerj-cs.1476 (PMC10496001; doi:10.7717/peerj-cs.1476)
Supplement: Supplemental Information 2 — The raw SUS and QUIS usability scores for the Cube and Grid interfaces. This also includes the detailed demonstration view, search tasks and queries used for experimental purposes. [file peerj-cs-09-1476-s002.zip › I-Cuboid Evaluation Material/I-Cuboid Evaluation Material/Tasks/Wildlife.docx]

**Wildlife**

**Grid Presentation**

**Task 1:**

**Steps:**

1. Input query “Indian Tiger”.
2. Select third image in twenty first row from the grid.
3. View details of the selected image.
4. Download the selected image on your computer.
5. View the neighbors (left, right, top, and bottom) of the selected image.
6. Scroll down till the end of the grid and Click on the next button.
7. On the second page, select third image in seventh row from the grid.
8. View details of the selected image.
9. Download the selected image on your computer.

**Task 2:**

**Steps:**

1. Input query “Indian Tiger”.
2. Identify an image of tiger in white frame, view its details, and download it on your computer from first page.
3. Explore the neighbor images of the selected image.
4. Select the next page by clicking the next button.
5. Identify gray scale image of white tiger walking in stones, view its details, and download it on your computer from second page.
6. Explore the neighbor images of the selected image.

**Wildlife**

**Cuboid Presentation**

**Task 1:**

**Steps:**

1. Input query “Indian Tiger”.
2. Select fifth plane from the cube.
3. Select third image in fifth row of fifth plane from the cube.
4. View details of the selected image.
5. Download the selected image on your computer.
6. View the neighbors (left, right, top and front) of the selected image.
7. Select any image from the neighbor images.
8. View details of the selected image.
9. Download the selected image on your computer.

**Task 2:**

**Steps:**

1. Input query “Indian Tiger”.
2. Identify an image of tiger in white frame by exploring all the planes of the cube, view the details of the identified image and download it on your image.
3. Explore the neighbor images of the selected image.
4. Select the next cube by clicking the next button.
5. Identify gray scale image of white tiger walking in stones, view the details of the identified image and download it on your computer.
6. Explore the neighbor images of the selected image.
